# Weighted Random Forests to Improve Arrhythmia Classification

**DOI:** 10.3390/electronics9010099

**Published:** 2020-01-03

**Authors:** Krzysztof Gajowniczek, Iga Grzegorczyk, Tomasz Ząbkowski, Chandrajit Bajaj

**Affiliations:** 1 Department of Artificial Intelligence, Institute of Information Technology, Warsaw University of Life Sciences - SGGW, 02-776 Warsaw, Poland; 2 Department of Physics of Complex Systems, Faculty of Physics, Warsaw University of Technology, 00-662 Warsaw, Poland; 3 Department of Computer Science, Institute for Computational Engineering and Sciences, University of Texas at Austin, Austin, TX 78712

**Keywords:** arrhythmia, false alarm, weighted random forest, machine learning

## Abstract

Construction of an ensemble model is a process of combining many diverse base predictive learners. It arises questions of how to weight each model and how to tune the parameters of the weighting process. The most straightforward approach is simply to average the base models. However, numerous studies have shown that a weighted ensemble can provide superior prediction results to a simple average of models. The main goals of this article are to propose a new weighting algorithm applicable for each tree in the Random Forest model and the comprehensive examination of the optimal parameter tuning. Importantly, the approach is motivated by its flexibility, good performance, stability, and resistance to overfitting. The proposed scheme is examined and evaluated on the Physionet/Computing in Cardiology Challenge 2015 data set. It consists of signals (electrocardiograms and pulsatory waveforms) from intensive care patients which triggered an alarm for five cardiac arrhythmia types (Asystole, Bradycardia, Tachycardia, Ventricular Tachycardia, and Ventricular Fultter/Fibrillation). The classification problem regards whether the alarm should or should not have been generated. It was proved that the proposed weighting approach improved classification accuracy for the three most challenging out of the five investigated arrhythmias comparing to the standard Random Forest model.

## 1. Introduction

Aggregation of machine learning based models is usually done by so called ensemble supervised learning [1]. The goal of ensemble algorithms is to combine the predictions of several base models built with a given learning algorithm in order to improve robustness and generalizability over a single model [2]. There is a strong evidence, that a single model can be outperformed by an ensemble of models combined to reduce bias, variance or both [3]. A single model is unlikely to capture the entire underlying structure of the data to achieve optimal predictions. This is where integrating multiple models can significantly improve prediction accuracy. By aggregating multiple base learners (individual models), more information can be captured on the underlying structure of the data [3].

Ensembling models constitute a relevant function in data analytics and can be created in a variety of ways. The three most popular methods for combining the predictions from different models are:

- Bagging or averaging aimed at building multiple models (typically of the same type) from different subsamples of the training dataset. The driving principle is to build several estimators independently (Bagging methods [4] and Random Forests [5]) and then to average their predictions;
- Boosting aimed at building multiple models (also typically of the same type) in a sequence. Each model learns to fix the prediction errors of a prior/preceding model (e.g., AdaBoost [6] and Gradient tree Boosting [7]). Base estimators are built sequentially and in each step the last one added tries to reduce the bias of the combined estimator;
- Voting (also called stacking) aimed at building multiple models (typically of different types). Uses simple statistics (like calculating the mean) to combine predictions [4]. It is also possible to take the output of the base learners on the training data and apply another learning algorithm on them to predict the response values [8].

Each of the aforementioned methods has different characteristics. Bagging tends to reduce variance and does not work well with simple machine learning models. Boosting reduces bias by sequentially combining weak learners but is sensitive to noisy data and outliers and is susceptible to overfitting [3]. Whilst voting/stacking reduces bias by fixing the errors that base learners made by fitting one or more meta-models on the predictions made by base learners. [3,8].

As previously stated, the construction of an ensemble model is a systematic process of combining many diverse base predictive learners. When aggregating predictive learners, there is always the question of how to weight each model and how to tune the parameters of the weighting process. The most straightforward approach is simply to average the base models i.e., to give equal weight to each base model. However, numerous studies have shown that a weighted ensemble can provide superior prediction results to a simple average of models [3,9–11]. There have been several attempts in the past to improve upon the classic version of the Random Forest (RF). Attempts to improve the accuracy of classification can be broadly divided into two categories:

- Pruning of individual trees in the forest [12,13];
- Weighing individual trees [11,14–17].

Therefore, this research is focused on application of some improvements to Random Forest (RF) algorithm, especially those dedicated to development of novel weighting algorithm applicable for each tree in the Random Forest model and the comprehensive examination of the optimal parameter tuning. The tunable parameters in the proposed approach include stability of the models, error on the unseen sample, and the parameter responsible for weights distribution. Such a broad set of parameters and the approach itself are motivated by its flexibility, stability of the performance, ability to capture non-linear dependencies, and resistance to overfitting that can deliver increased predictive performance. To evaluate the proposed weighting approach, numerical experiments on five data sets regarding arrhythmia classification have been conducted. To demonstrate the generalizability of the proposed scheme we compare achieved results to the results obtained in our previous study (standard version of Random Forest) [18] examining data provided by organizers of the Physionet/Computing in Cardiology Challenge [19]. During the challenge, participants were provided with 750 signals registered 5 min before the alarm generation and information about what type of arrhythmia caused the alarm. The alarms were triggered by five types of arrhythmia: Asystole, Bradycardia, Tachycardia, Ventricular Fibrillation or Flutter, and Ventricular Tachycardia. All the signals were analyzed by expert annotators and labelled as true or false [18,19].

The main questions that we want to address in this paper are:

- Does the proposed weighting method introduce improvements to the standard Random Forest algorithm?
- To what extent is it possible to outperform previous results of reducing false arrhythmia alarms?
- What is the effect of different tuning parameters as part of finding optimized ensembles on the quality of predictions?
- Can the results be generalized over different arrhythmia types (datasets of different characteristics)?

The remainder of this paper is organized as follows: Section 2 provides an overview of the similar research problems for predictive models aggregation, weighting schemas, and arrhythmia classification. In Section 3, the theoretical framework of the weighted Random Forest algorithm is presented. In Section 4, the research framework was outlined, including the details of numerical implementation, feature vector description and model performance measures. Section 5 outlines the experiments and presents the discussion of the results. The paper ends with concluding remarks in Section 6.

## 2. Literature Review

There have been extensive studies on weighted ensembles in the literature. Shahhosseini et al. [3] propose a systematic approach to find the optimal weights to create ensembles for bias-variance tradeoff using cross-validation for regression problems (Cross-validated Optimal Weighted Ensemble (COWE)). It is known, that tuning hyperparameters of each base learner during the ensemble weight optimization process can produce better performing ensembles. Therefore, authors proposed a nested algorithm based on bi-level optimization that considers tuning hyperparameters as well as finding the optimal weights to combine ensembles (Cross-validated Optimal Weighted Ensemble with Internally Tuned Hyperparameters (COWE-ITH)). Later on, Pham et al. [14] proposed a weighted scheme that generalizes bagged ensemble learning to a weighted vote by considering different ways of averaging. Kuncheva et al. [20] proposed a probabilistic framework for classifier combination. It gives rigorous optimality conditions (minimum classification error) for four combination methods: majority vote, weighted majority vote, recall combiner, and the naive Bayes combiner. The framework is based on two assumptions: class-conditional independence of the classifier outputs and an assumption about the individual accuracies. Filmus et al. [21] analyzed the weighted voting games (WVGs) i.e., a class of cooperative games that capture settings of group decision making in various domains, such as parliaments or committees.

In Breiman’s [5] forests, the final prediction is the average of the individual tree outcomes. A natural way to improve the method is to incorporate tree-level weights to emphasize more accurate trees in prediction. The concept of weighted trees in the Random Forest is not entirely new. Winham et al. [11] described the weighted Random Forest method, which incorporates tree-level weights into the usual RF algorithm to emphasize more accurate trees in prediction and calculation of variable importance. They considered different tree-weights, and present simulations to compare the performance of the proposed approach to the traditional RF algorithm both in terms of prediction accuracy and performance of variable importance measures. Pham et al. [22] described a potential improvement on the Random Forest (for the binary classification problem) using Cesaro average. This method is motivated by the potential instability of averaging predictions of trees. Byeon et al. [15] used Out-of-Bag (OOB) samples for deriving Akaike weights while averaging the tree results. Based on the proposed algorithm they analyzed in-depth the consumers’ demand level in order to operate “the Voucher Program for Speech Language Therapy” efficiently and for suggesting different ways to improve the special education service support. Xuan et al. [16] introduced Refined Weighted Random Forests (RWRF) to credit card fraud detection. The improvement regards two aspects. They used all training data (including In-Bag (INB) data and OOB data) because performance evaluation of different base classifiers should have the same evaluation dataset. Moreover, they used margin between probability of predicting true class and false class label which measures the extent to which the expected number of votes for the right class exceeds the expected number of votes for other class. Kulkarni et al. [17] presented attempts to improve performance of Random Forest classifier in terms of accuracy, and time required for learning and classification. They are based on disjoint partitions of training datasets, use of different attribute evaluation/split measures to induce base decision trees of Random Forest, application of weighted voting instead of majority voting, use of diversity among bootstrap datasets to generate maximum diverse classifiers, and application of dynamic programming approach to find optimal subset of Random Forest.

Moreover, there are multiple, different application areas in which weighted ensemble approaches (especially Random Forests) are used. Booth et al. [23] introduced an automated trading system based on performance weighted ensembles of Random Forests that improves the profitability and stability of trading seasonality events. The results show that recency-weighted ensembles of Random Forests produce superior results, in terms of both profitability and prediction accuracy, compared to other ensemble techniques. Utkin et al. [24] proposed weighted Random Survival Forest which can be regarded as a modification of the Random Forest improving its performance. The main idea underlying the proposed model is to replace the standard procedure of averaging used for estimation of the Random Survival Forest hazard function by weighted averaging. The weights are assigned to every tree and can be viewed as training parameters which are computed by solving a standard quadratic optimization problem maximizing Harrell’s C-index. Finally, Sunil Babu et al. [25] utilized an effective meta-heuristic feature selection technique along with hybrid Naive Bayes (NB) and sample weighted Random Forest (SWRF) classification approach for sub-acute ischemic stroke lesion segmentation. Here the NB classifier is trained and applied to estimate the weights of training samples. Then, the training samples with estimated weights are utilized to train SWRF. Brief comparison of the weighting methods applied for ensemble classifiers is presented in Table 1.

Automatic detection and classification of life-threatening arrhythmia plays an important role in dealing with various cardiac conditions. An arrhythmia occurs when the heartbeat is irregular. Some arrhythmias are defined only by the frequency of heart contractions. Bradycardia occurs when the number of heartbeats is less than 40 per minute (bpm). Tachycardia is diagnosed when heart rate exceeds 140 beats per minute. The Asystole occurs where there are no heart contractions detected for at least four seconds. Ventricular Flutter or Fibrillation and Ventricular Tachycardia are arrhythmias that not only have an abnormally fast rhythm, but the morphology of QRS complexes differs from normal. Such precise definitions make arrhythmias easy to detect from an algorithmic point of view, but only when high quality data is available. In real life, signals are often noisy and contain artifacts, which is why it is so important to use robust algorithms to locate the heartbeats within the signal.

To detect and to analyze Bradycardia, Tachycardia, and Asystole the key is to correctly identify locations of QRS complexes (or in terms of Asystole, lack of them). The electrocardiography (ECG) signal is the main source of information about the patient’s cardiovascular condition, but because the measurements are taken from the patient’s body motion artifacts, sweating or muscle spasms can impact the signal. Therefore, particular attention should be paid to assessing the quality of the data. In bedside monitors, in addition to ECG, pulsatile waveforms such as blood pressure (BP) or plethysmogram (PLETH) are also recorded. Such recordings may provide necessary information about the heart rate when ECG signal quality is very low. In the first row of Table 2, we present methods commonly used for detection of QRS complexes. First, are methods based on ECG signal analysis only and then algorithms using supplementary information gathered from other medical signals (e.g., blood pressure) as well. Next, there is a section dedicated to each type of arrhythmia and the methods of diagnosing it (if applicable). The last row of the table contains more general methods used to classify all types of arrhythmias. They were used with a different parameter configuration for each arrhythmia.

## 3. Weighted Random Forest

Let’s consider a binary classification problem, having a training sample (***D***) of n(i∈{1,…,n}) observations, a class variable ${ϒ}_{i}\in \{0,1\},$ and *p* predictor variables $X_{1},\ldots ,X_{p}(k\in \{1,\ldots ,p\}).$ The main objective is to find a model for predicting the values of *Y*_*i*_ from new ***X*** values [43].

Random Forest algorithm incorporates the bagging procedure to each decision tree (base learner) [33], generating *Ntree* (*j* ∈ {1,...,*Ntree*}) new training datasets ***D***_*j*_ (also called In-bag sample; further denoted as *INB*), each of size $n^{\prime }$ by sampling from ***D*** uniformly and with replacement (this causes that some observations might be duplicated). Each time In-bag sample is expected to have approximately $1-\frac{1}{e}\approx 63.2\%$ of the unique observations, while rest of them fall into the so called Out-of-Bag sample (further denoted as *OOB*). The RF algorithm has an additional sampling stage (called Random Decision Tree) selecting a random subset of the features *mtry* at each candidate split in the learning process. Typically, for a classification problem with *p* features, $floor(\sqrt{p})$ features are used in each split [52].

The original implementation of Random Forest aggregates tree-level results equally across trees obtaining the final probability ${\hat{ϒ}}_{i}^{RF}$ (so called score) for a given observation using one of the following formulas:

(1) $${\hat{ϒ}}_{i}^{RF}=\sum_{j=1}^{Ntree}I\left[{\hat{ϒ}}_{ij}>0.5\right],$$

where *I* is the indicator function, ${\hat{ϒ}}_{ij}$ is the probability for *i*-th observation assigned by the *j*-th tree. The above formula uses majority voting with predefined threshold set at 0.5, which sometimes might not be an appropriate approach. Therefore, to gain some flexibility (and to be more sensitive for any possible deviations from the desired output), in this article the following formula is used:

(2) $${\hat{ϒ}}_{i}^{RF}=\frac{1}{Ntree}\sum_{j=1}^{Ntree}{\hat{ϒ}}_{ij},$$

which is in other words a simple average of the probabilities from each tree.

In this research, we implemented the usual RF algorithm to build the trees of the forest, however, we utilize performance-based weights for tree aggregation. In particular, we considered weighting probability from each tree in the forest, such that better performing trees are weighted more heavily:

(3) $${\hat{ϒ}}_{i}^{RF}=\frac{1}{Ntree}\sum_{j=1}^{Ntree}{\hat{ϒ}}_{ij}*w_{j}.$$

Because weights are based on the performance of the particular/a distinct tree, applying the weights to the same dataset from which they were calculated (as was done in [38,53]) would bias prediction error assessment. Therefore, estimates of the predictive ability of each tree are calculated using both the *INB* and the *OOB* observations, which can be further used to calculate weights. In order to obtain trees having good generalization abilities, we introduced a special tree performance measure (*θ*) incorporating weighted Area Under the Curve (AUC). Trees having the greatest values of the following measure should have higher weights (in Equation (3)) while determining the final probability:

(4) $$\theta =f\left(AUC_{INB},AUC_{OOB}\right)=-\alpha \left|AUC{\left(w^{obs}\right)}_{INB}-AUC_{OOB}\right|+(1-\alpha )AUC{\left(w^{obs}\right)}_{OOB^{\prime }}$$

where *AUC*_*INB*_ stands for the In-Bag sample accuracy, *AUC*_*OOB*_ for the Out-of-Bag sample accuracy and $w^{obs}$ is an observation level weight vector (please see next paragraph). Parameter *α* measures the weights of the first and the second term in the equation i.e., it controls what is more important during learning, stability of the tree, or small errors on the unseen dataset [54].

It should be noticed that some observations are more difficult to correctly classify than others. That is why some algorithms incorporate observation weighting. A classic example is the AdaBoost algorithm (learning in iterative/sequential manner) where for each iteration the observation weights are individually modified and the classification algorithm is reapplied to the weighted observations. At each step, those observations that were misclassified by the model induced at the previous step have their weights increased, whereas the weights for those classified correctly are decreased. Thus, as iterations proceed, observations that are difficult to be classified correctly receive ever-increasing influence. Each successive model is thereby forced to concentrate on those training observations that are missed by previous ones in the sequence [55].

Observation weighting can be also considered as a fairness problem [56]. By definition, fairness is the absence of any prejudice or favoritism towards an individual or a group based on their intrinsic or acquired traits in the context of decision-making [56]. Thus, an unfair algorithm is one whose decisions are skewed toward a particular group. For instance, in Random Forest some trees might have better performance on an OOB sample because they were trained on the similar observations (INB sample). Such observations are hard to predict correctly by the trees that did not have them in INB used for training.

In this article, we used this idea for estimating the performance of each tree. Weighted AUC can handle such weights implicitly so instead of using 1 for a given example, it uses the specified weight $w_{i}$ derived based on the observations in Out-of-Bag sample:

(5) $$w_{i}^{obs}=\frac{1}{\#OOB}\sum_{j\in OOB}\left|{ϒ}_{ij}-{\hat{ϒ}}_{ij}\right|.$$

If for instance $w_{i}^{obs}=0$ (which means that each tree correctly predicted the class) the example is practically ignored. As a result, the miss-classified examples have more influence on the final performance of a given tree.

In order to estimate weighted AUC (Formula (4)), as we assumed ${\hat{ϒ}}_{i}\in \mathbb{R}$ is the predicted score for each observation, let $\Gamma_{1}=\left\{i:Y_{i}=1\right\}$ be the set of positive examples and $\Gamma_{0}=\left\{i:Y_{i}=0\right\}$ be the set of negative examples. Then, the total positive weight is $W_{1}=\sum_{i\in \Gamma_{1}}w_{i}^{obs}$ and the total negative weight is $W_{0}=\sum_{i\in \Gamma_{0}}w_{i}^{obs}$ [57]. Moreover, for any threshold τ∈ℝ, we defined the thresholding function $t_{\tau }:\mathbb{R}\to \{0,1\}$ such as:

(6) $$t_{\tau }(\hat{ϒ})=\{\begin{array}{l} 1,\hat{ϒ}\geq \tau \\ 0,\hat{ϒ}<\tau \end{array}.$$

Based on the above formula the weighted false positive rate is defined as:

(7) $$FPR(\tau )=\frac{1}{W_{0}}\sum_{i\in \Gamma_{0}}I\left[t_{\tau }\left({\hat{ϒ}}_{i}\right)\neq 0\right]w_{i}^{obs},$$

where *I* is the indicator function that is 0 for a correct prediction, and 1 otherwise. On the other hand, the weighted the true positive rate is defined as:

(8) $$TPR(\tau )=\frac{1}{W_{1}}\sum_{i\in \Gamma_{1}}I\left[t_{\tau }\left({\hat{ϒ}}_{i}\right)=1\right]w_{i}^{obs}.$$

A weighted ROC curve is drawn by plotting *FPR*(*τ*) and *TPR*(*τ*) for all thresholds τ∈ℝ. Finally, the weighted AUC $\left(AUC\left(w^{obs}\right)\right)$ measure can be calculated using the trapezoid rule (integral), which is known to be extremely accurate when approximating the definite integral of periodic functions [57].

Next, a question arises: how much greater should be weights for the best trees? In the literature, there are several approaches proposed to determine weights in multi-criteria decision making [58]. One of them incorporates ranked weights. The rank order weight determination is comprised of two stages:

- Ranking the pre-defined criterion (*θ*) according to their importance (performance of the tree derived using Formula (4));
- Weighting the criteria from their ranks using some rank order weighting approach.

In other words, let’s assume having a list of *Ntree* prioritized (ranked) criteria, where each criterion *j* has a rank *r*_*j*_ (*j* = 1,...,*Ntree*). The goal is to select and rank a set of *Ntree* criteria that seems to be relevant, giving each *j*-th criterion a rank *r*_*j*_. The rank is inversely related to weight, which means that first rank *r*_1_ = 1 denotes the highest weight (best tree), whilst rank *r*_*Ntree*_ = *Ntree* denotes the lowest weight (worst tree). Many authors suggest various approaches for assigning weights based on a given criterion e.g., rank reciprocal (inverse), rank sum (linear), and rank exponent weights [59]. In this paper, we assumed that weights should be exponential [58]:

(9) $$w_{j}=\frac{{\left(Ntree-r_{j}+1\right)}^{p}}{\sum_{k=1}^{Ntree}{\left(Ntree-r_{k}+1\right)}^{p}{}^{\prime }}$$

**Figure 1.**
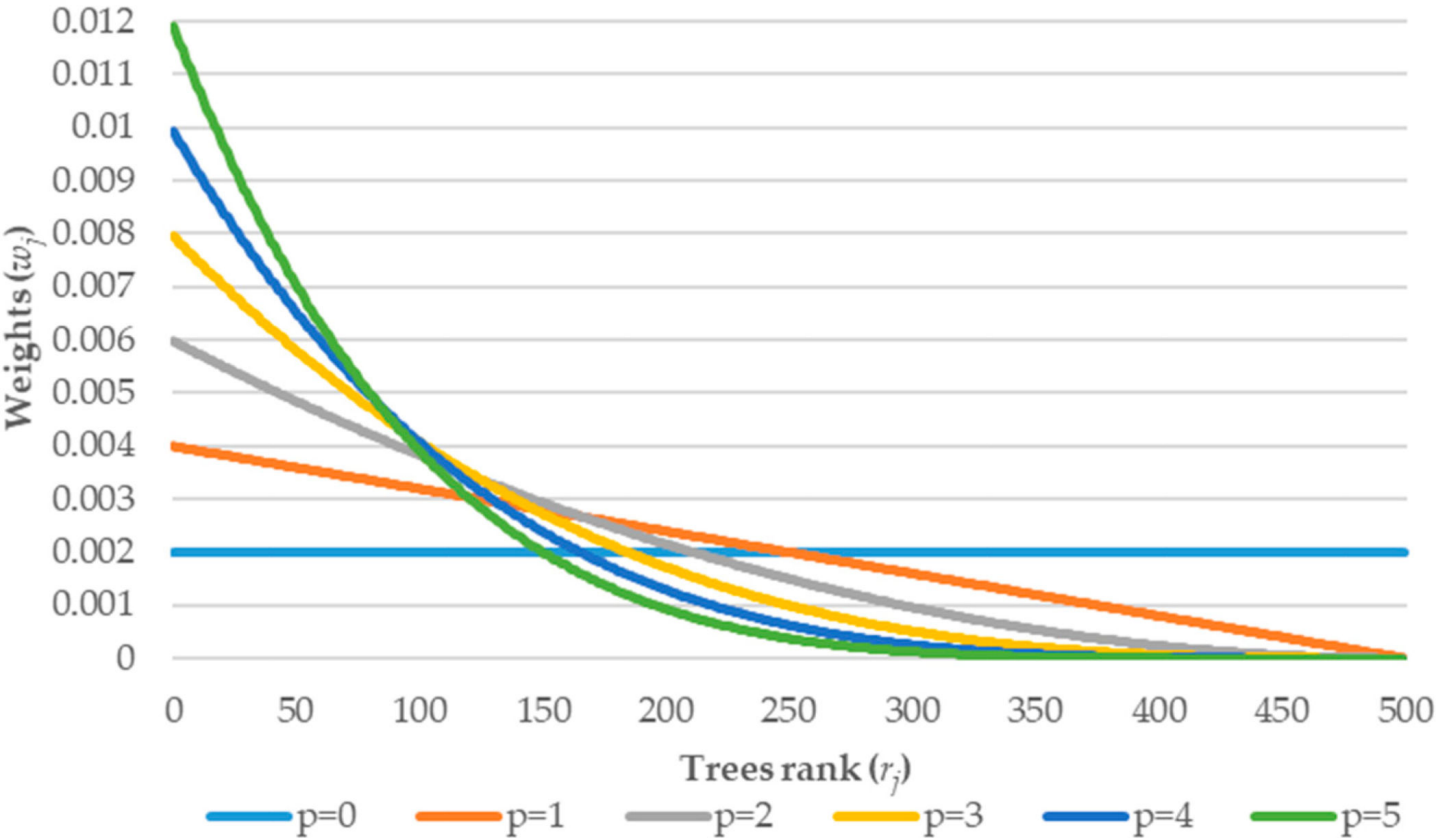
Weights distribution in terms of number of trees and value of the parameter *p*.

Where *r*_*j*_ is the rank of the *j*-th tree, *p* is the exponential parameter describing the strength of the weights. All weights are normalized and, in consequence, sum up to 1. An example of weights’ influence on the final probability (Equation (3)) inducted by the parameter *p* is presented in Table 3 and Figure 1 below.

In this example, the best performance in terms of Equation (4) was the third tree (fourth column). Because parameter *p* is set to 2 the nominator of the exponential rank weight equals 16 (4 − 1 + 1)^2^) and after the normalization (by denominator) 0.533. Analyzing Figure 1, it can be stated that when parameter *p* equals 0 it is standard aggregation, where each tree has the same weight. Whilst *p* = 1 represents linear rank sum weight. When *p* increases, the weights distribution becomes steeper, i.e., the greater the parameter *p* is the final probability is more influenced by the trees having better performance.

Like any weighting scheme, determining where to apply a particular weight is of utmost importance. With a Random Forest with *j* (usually 500)trees, *mtry* features used in each split (here *floor* $\left(\sqrt{p}\right))$ and ***D*** training dataset the complete Weighted Random Forest algorithm pseudocode is summarized in Algorithm 1 (below).

**Algorithm 1.** Weighted Random Forest algorithm pseudocode.

**input:** Number of Trees (*Ntree*), random subset of the features (*mtry*), training dataset (***D***)
**output:** Random Forest (***RF***)
1: ***RF*** is empty
2: **for each** *j* to *Ntree* **do**
3: ***D***_*i*_ = Bootstrap Sample (***D***)
4: ***DT***_*i*_ = Random Decision Tree (***D***_*i*_, *mtry*)
5: ***RF*** = ***RF*** ∪ ***DT***_*i*_
6: **end**
7: **for each** *i* to *n* **do**
8: Compute $w_{i}^{obs}$ using Formula (5)
9: **end**
10: **for each** *j* to *Ntree* **do**
11: $\theta_{j}=f\left(AUC{\left(w^{obs}\right)}_{INB_{j}},AUC{\left(w^{obs}\right)}_{OOB_{j}}\right)$
12: **end**
13: **for each** *j* to *Ntree* **do**
14: Compute $w_{j}$ using Formula (9)
15: **end**
16: **for each** *i* to *n* **do**
17: Compute final prediction ${\hat{ϒ}}_{i}^{RF}$ using Formula (3)
18: **end**
19: **return *RF***

Our implementation may suggest that this algorithm is applicable to Random Forest only. However, it can be generalized to any ensemble consisting of any kind of *j* base models. Which is done by changing observations weighting based on the Out-of-Bag samples (Formula (5), line 8 in the pseudo-code above) by any other sample e.g., training dataset.

## 4. Research Framework and Settings

### 4.1. Feature Vector

Database used in this paper consisted of 750 multi-signal recordings registered for patients on Intensive Care Unit (ICU). Registered signals were 5 min in length (sampling frequency 250Hz) and ended with an alarm generated for one of five types of arrhythmia. Each of the recordings had two leads of the ECG, at least one pulsatile waveform (arterial blood pressure (ABP) or photoplethysmogram (PLETH)) and respiratory signal. Distribution of the recordings among the investigated five types of arrhythmias and whether the alarm should or should not have been generated are: Asystole (No—100, Yes—22); Bradycardia (No—43, Yes—46); Tachycardia (No—9, Yes—131); Ventricular Tachycardia (No—252, Yes—89); Ventricular Fibrillation or Flutter (No—52, Yes—6). The signals provided were already pre-filtered with multiple notch filters and finite impulse response (FIR) band pass filter (0.05–40 Hz) [18,19].

As mentioned in Section 2, to diagnose Asystole, Bradycardia, and Tachycardia it is critical to properly locate consecutive heart beats. Hence, the first step to create features was the detection of QRS complexes in the ECG signal, performed by a low-complexity R-peak detector as described in [18,51]. At the same time, an open source *wabp* algorithm [19] was used to locate the beats in pulsatile waveforms provided (ABP, PLETH). The quality of the beats detection was assessed by comparing obtained QRS locations among the signals. Each located beat was marked as true positive (TP)—if it was found in both signals or false positive (FP) and negative (FN), respectively, in compared signals, depending on the order in which they were being compared [18,51]. Then, an F1-score as F1 = 2 * TP/(2 * TP + FP + FN) was calculated. The more beat locations were similar in the signals, the closer F1-score was to 1. If there were no matches of beat locations the F1-score equaled 0. The two signals with the highest F1-score were considered in the following analysis [18,51].

In diagnosing Ventricular Flutter or Fibrillation and Ventricular Tachycardia, the features were generated with spectral purity index (SPI) [44,51]. The reason why, these arrhythmias require a different method of detection of physiological QRS complexes as it is impossible due to the nature of ventricular originated arrhythmias (see Section 2). The maxima and minima of obtained SPIs were used as features to check if the alarm should have occurred i.e., max and min SPI for Ventricular Tachycardia and max SPI for Ventricular Fibrillation or Flutter.

### 4.2. Numerical Implementation

All numerical experiments presented below were prepared using *R* programming language [60] working on Ubuntu 18.04 operating system on a personal computer equipped with Intel Core i7–9750H 2.6 GHz processor (12 threads) and 32 GB RAM. The entire weighted Random Forest algorithm was built based on own modification of the following R libraries: *ranger*—implementing in in *C*++ and *R* the state-of-the-art Breiman’s RF, especially suited for high dimensional data [61]; *pROC*—for finding optimal threshold for class determining based on the Youden Index [62]; *WeightedROC*—for fast computation of Receiver Operating Characteristic curves and Area Under the Curve for weighted binary classification problems. Furthermore, to perform this study, many wrapper functions working on the output from the *ranger* package were written, e.g., functions extracting the class probability matrix or In-Bag/Out-of-Bag matrix for each observation and tree [18].

The estimates for the performance measures for the training and validation samples were produced with *k*-fold cross-validation [18]. The number of *k* sets was set to 10 when there were more than 10 samples in the smaller class. Otherwise, *k* was set to the size of the smaller class to ensure that there was at least one sample from both of the classes (e.g., six for ventricular fibrillation or flutter) [18]. The *k* sets were generated so that the class distribution in every set represented the class distribution of the entire dataset using stratified sampling based on the *createFolds* function implemented in the *caret* library [63]. All further results are presented as an average over *k*-folds.

### 4.3. Performance Measures

A proper evaluation is crucial for models built with any statistical learning algorithm. Hence, in this research different types of evaluation metrics were used. The main measure used for this purpose in the Challenge is the Score measure defined as:

(10) $$\text{Score}=\frac{100\times (TP+TN)}{TP+FP+TN+5\times FN^{\prime }}$$

where for a binary classification problem the following indications are used [64]: *TP* and *TN* denote number of correctly classified either positive or negative instances, *FP* stands for the instances predicted as Yes when the actual output was No, and finally *FN* indicate the number of instances predicted as No while the actual output was Yes. According to the above formula it can be seen that the measure was designed to treat *FN-false negative* (genuinely life-threatening events that the program considered unimportant) especially harshly [18,51].

The second used measure is Area Under the ROC Curve, which is particularly important in this research since it was used to tune the parameters of each model [18]. The construction of the ROC curve and the calculation of the AUC measure was described in Section 3 [52,54].

### 4.4. Benchmarking Methods

In order to compare and assess the quality of the proposed weighting approach, we treated results obtained in our previous study as a benchmark [18] i.e., the standard Random Forest, where final probability (requires AUC calculation) for each observation is derived using majority class voting with the cutoff set to 0.5 (Voting Prob 0.5) and the final class for each observation incorporates a cutoff set to 0.05 (Vote 0.5 0.5).

In addition to the above method, the following benchmarking algorithms have been used. First is the CART algorithm implementing classification and regression trees (*rpart* library) [65]. It utilizes pruning during the growth stage. In order to generalize the knowledge, this approach prevents new splits from being created when the previous splits provided only a slight increase in predictive accuracy. The complexity parameter *cp* varied from 0 to 0.1 in increments of 0.01.

The second model has been built based on the framework described in [66], in order to construct a model of the support vector machine, the C-SVM function from the *kernlab* library was used. The linear, polynomial (degrees 1, 2, and 3) and radial (*γ* from 0.1 to 1, by 0.2) kernel functions were used and *ε* (which determines the margin width for which the error function is zero) was arbitrarily taken from the following set of {0.1, 0.3, 0.5, 0.7, 0.9}. The regularized parameter *C* that controls overfitting has been arbitrarily set to one of the following values {0, 0.2, 0.4, 0.6, 0.8, 1}.

The last results were derived based on the AdaBoost algorithm implemented in the *adabag* library. In this case, the number of iterations for which boosting is run (or the number of trees to use) has been set to 100 iterations, the weight updating coefficient *α* has been set to 1/2*ln*((1 − *error*)/*error*), and finally each tree was at most 5 levels deep.

Finally, in each tuning parameter process, a structure maximizing the Score function (Equation (10)) on the training dataset has been chosen as a final structure of the model.

### 4.5. Tuning of the Weighting Parameters

To answer the question about the effect of the tuning of the weighting parameters in terms of the quality of predictions we performed a grid search checking various combinations of the *α* and *p* parameters:

To check the effect of the weighting parameters tuning, in terms of predictions quality, we performed a grid search checking various combinations of α and *p* parameters:

- *α* ∈ {0, 0.1, 0.2, 0.3, 0.4, 0.5, 0.6, 0.7, 0.8, 0.9, 1}—controlling the importance of the first (model stability) or the second (small error on the unseen dataset) term in Formula (4).
- *p* ∈ {0, 0.5, 1, 1.5, 2, 2.5, 3, 3.5, 4, 4.5, 5}—which is the exponential parameter describing the strength of the weights (distribution).

It would purely be an explanatory analysis, giving an insight into investigated phenomena.

## 5. Empirical Analysis

The classification performance of the proposed approach was evaluated with AUC and a Challenge Score (Formula (10)) within different datasets (In-Bag, Out-of-Bag, Training, and Validation). In the tables below, we present exemplary results, as values obtained for Validation dataset (Tables 4–8). This dataset was chosen as representative, since the Validation sample did not take part during the model training. The results were calculated as average values based on *k*-cross validation.

Each table first presents results for AUC measure and then for the Score obtained using standard Random Forest algorithm (column Base). The next column includes different values of the parameter *α* and the headings of the subsequent columns show values of the parameter *p*. Finally, intersections of consecutive values of *α* and *p* parameters present/demonstrate improvement (or deterioration) for all aforementioned measures. It should be noted that *p* = 0 leads to equal weights (as in case of standard Random Forest). For quick identification of desired/undesired combinations we set a proper color scale i.e., improvements are marked in (light) green while deterioration is marked in (light) orange.

In Table 4, the AUC for Asystole based on the standard Random Forest is 0.93. Applying weighted version results with an improvement of 0.055 from most of the combinations gives an AUC of 0.985. In the bottom-right corner we can see an even greater improvement of 0.060. For this type of arrhythmia, Score values show constant behavior with an improvement of 19.00 units (61.67 + 19 = 80.75). For Bradycardia, we can observe diverse results for both AUC and Score (Table 5). The left-bottom corner for the AUC presents intersections where applying weights can improve results (approximately 0.001). Other regions of the table show a slight performance deterioration for both measures.

Results for Tachycardia (Table 6), in terms of AUC, present either infinitesimal improvement, for all ranges of *α* when the exponential parameter is less than about 2.5 or slight deterioration of approximately 0.008 while increasing the alpha to above 2.5. Score is constant except for *p* = 5 and *α* = 0.6.

The AUC for the base RF model for Ventricular Fibrillation or Flutter reaches 0.97 (Table 7). Applying weighted Random Forest resulted in almost ideal classification, since the measure is above 99.5 for all combinations of the tuned parameters. Such improvement influences also Score measure which increases of 55.55 for most intersections (some combinations boosted results of 54.51). Results for Ventricular Tachycardia (Table 8) shows that the best results for the Score can be obtained when weights vanish linearly (*p* equals 1) for alpha parameters less than 0.9. Slightly worse results can be obtained while using *α* between 0.7 and 1 and *p* between 2.5 and 4.5 (middle-bottom part of the Table 8).

As presented in Table 9, there are three types of arrhythmia i.e., Asystole, Ventricular Tachycardia, and Ventricular Fibrillation or Flutter which are difficult to predict, when looking into the results for the validation dataset. However, application of weighted RF resulted in improved classification. Importantly, the performance of weighted RF models in terms of the Score measure observed on the validation sample confirms that the method works well and is able to capture arrhythmias with high accuracy. The following Scores were obtained for the proposed approach:

- 31.9 for Ventricular Tachycardia—accuracy of the model was improved in comparison to RPART (27.7), C-SVM (30.5), and AdaBoost (29.9);
- 86.1 for Ventricular Fibrillation or Flutter—accuracy of the model was improved in comparison to RPART (29.4), C-SVM (50.1), and AdaBoost (50.1);
- 80.7 for Asystole—accuracy of the model was improved in comparison to RPART (52.1), C-SVM (61.7), and AdaBoost (61.7).

**Figure 2.**
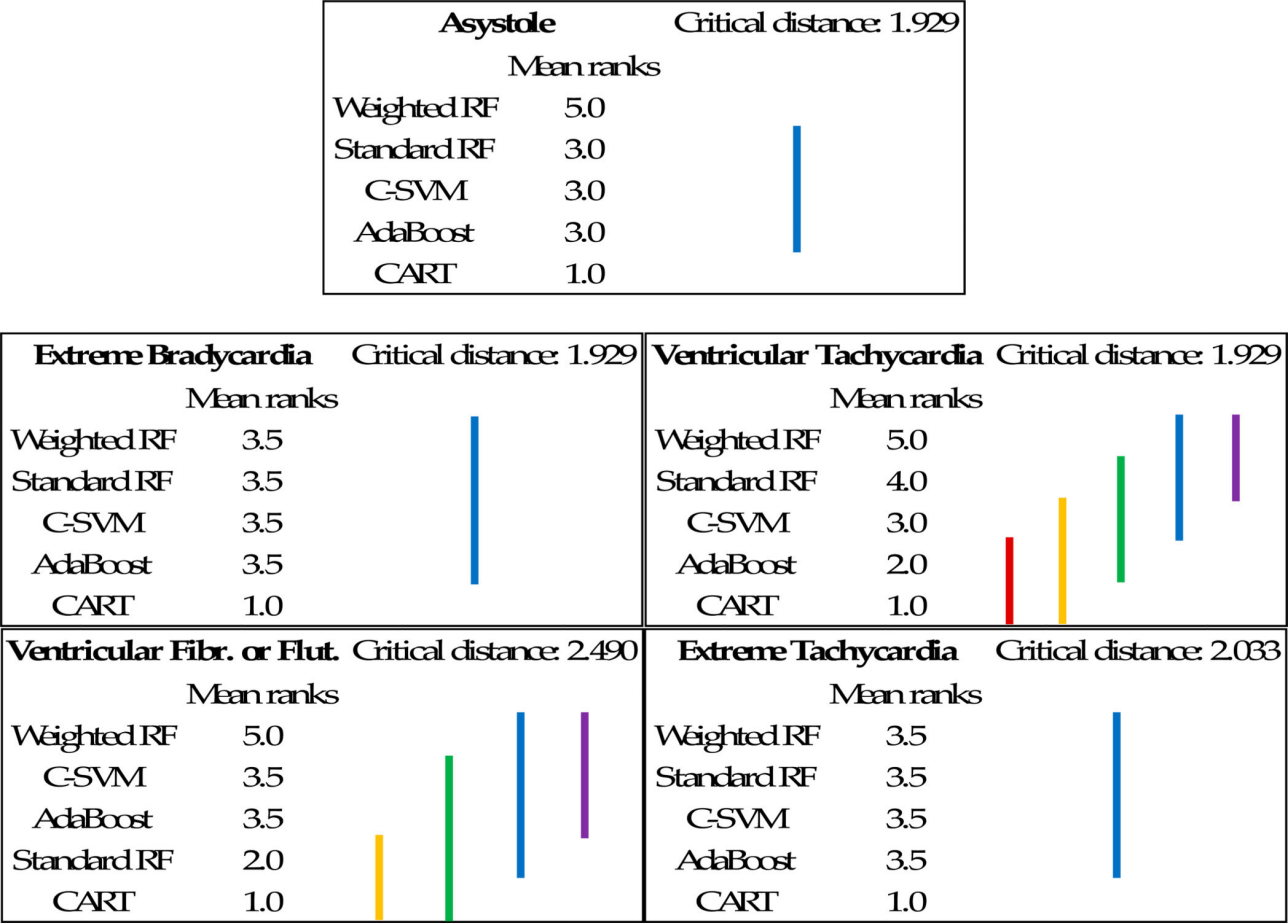
Nemenyi diagram for benchmarking methods for Score measure.

Finally, in order to assess whether differences in Score measures for all methods presented in Table 9 are statistically significant, we have used Nemenyi post-hoc tests [67] (test is performed by taking into account each of the *k*-folds). This is a post-hoc test intended to find the groups of data that differ after a statistical test of multiple comparisons (such as the Friedman test) has rejected the null hypothesis that the performance of the comparisons on the groups of data is similar. The test makes pair-wise tests of performance. Moreover, for a better understanding of the results the Critical Difference (CD) diagram depicted in Figure 2 has been created. This diagram provides an interesting visualization of the statistical significance of the observed paired differences between a set of models on a set of different datasets (i.e., dataset for each arrhythmia type). It allows one to compare all of the models against each other on these datasets and check the results of all these paired comparisons.

In the resulting diagram, each model is represented in rows (with an average rank position across *k*-folds). The null hypothesis is that the average ranks of each pair of models do not differ with statistical significance (at confidence level *α* = 0.95). Vertical lines connect the models for which we cannot exclude the hypothesis that their average ranks are equal. Any pair of models whose lines are not connected can be seen as having an average rank that is different with statistical significance. On top of the graph, the critical difference depicts the required difference between the average ranks for the two pair of models to be considered significantly different.

Based on the Nemenyi diagram, it can be stated that improvement in results for Asystole are statistically significant in comparison to all the benchmarking methods. For Ventricular Tachycardia, despite the slight improvement in this measure (see Table 9), the difference between standard and weighted RF is insignificant. On the other hand, Random Forests give statistically significant better results than other methods. Comparing standard and weighted RF it can be see that results improvement is statistically significant for Ventricular Fibrillation or Flutter.

The results indicate that weighting applied to Random Forests can be considered a viable approach aimed at improvement of the classification especially in case when the benchmarking methods like decision trees, while support vector machines are not able to deliver acceptable accuracy.

## 6. Conclusions

Over recent years, the idea to aggregate the machine learning; based models has been extensively studied and documented in the literature. Researchers have often shown that combining the predictions of several base models, built with a given learning; algorithm, improves robustness and generalizability over a single model. Ensemble models such as Bagging;, Boosting, Random Forests, and stacking have different characteristics. Some approaches do not work well with relatively simple models or are sensitive to noisy data and outliers.

While aggregating predictions from the base models, there is always a question whether better models should have greater influence on the final performance. The easiest way is to treat all predict^i^ons equally while more complex approaches assign bigger weights to superior models. The comprehensive ensemble framework should simultaneously tune various parameters (including stability of the models), error on the unseen sample and the parameter responsible for weight distributions. Therefore, the motivation of this study was to propose the new weighting algorithm applicable for standard Random Forest algorithm and the comprehensive examination of the optimal parameter tuning. To demonstrate the generalizability of the proposed scheme we conducted the study based on the Physionet/Computing in Cardiology Challenge, 2015. The Challenge addresses the issue of false arrhythmia alarms in the Intensive Care Units, which has detrimental consequences for both patients and medical staff. The analyzed dataset consists of medical signals (ECG, BP, and PLETH) for which triggered arrhythmia al arms and the classification problem is to solve whether the generated alarm was true of false. It is a challenging task for any classification algorithm as there could be a number of triggers for false alarms, e.g., the noises and machine malfunctions affecting the; signals and in consequence highly influencing the model performance.

The novelty of the research was demonstrated through weighting algorithm design to enhance the Random Forest model. It proves that the proposed solution is robust and can deal not only with easily diagnosable arrhythmias like Asystole or Bradycardia but also with arrhythmia types which are difficult to predict, i.e., Ventricular Tachycardia and Ventricular Flutter and Fibrillation. The results proved that the weighted Random Forest is able to detect three arrhythmias (i.e., Ventricular Tachycardia, Ventricular Flutter and Fibrillation, and Asystole) with better accuracy than the benchmarking standard Random Forest. In the case of Bradycardia and Tachycardia, after proper tuning of the parameters, the classification accuracy can be slightly improved too. Although, it is important to acknowledge, that the accuracy of Bradycardia and Tachycardia detection is already high, as these are arrhythmias without any morphological changes in the ECG signal, which makes them relatively easy to predict. Finally, comparing our proposed algorithm with three state-of-the-art benchmarking methods one can see that for Asystole and Ventricular Tachycardia results improvements are statistically significant.

The authors believe that the problem to reduce the number of false alarms, while avoiding the suppression of true ones, is valid and therefore, the study can be extended further with the application of other other weighting approaches to obtain higher classification accuracy. This may lead to further study on algorithms’ diversity and their effects on performance.

Future work in this area should include extending this weighted ensemble framework to multiclass classification and a regression problem. Secondly, applying a similar concept on other ensemble creating methods. We intend to further explore the performance and default parameter settings in the context of the bias and variance of the base classifier, with potentially both a theoretical and empirical analysis.

## Figures and Tables

**Table 1.** Weighting methods for ensemble classifiers in the literature.

Work	Method Applied	Conclusion
[11]	Tree-level weights in Random Forest.	Method does not dramatically improve predictive ability in high-dimensional genetic data, but it may improve performance in other domains.
[14]	Tunable weighted bagged ensemble using CART, Naïve Bayes, KNN, SVM, ANN and Logistic Regression.	Approach can usually outperform pure bagging, however, there are some cons in terms of time considerations in effectively choosing tunable parameters aside from a grid search.
[15]	Variable importance-weighted Random Forest.	Better prediction power in comparison to existing random forests granting the same weight to all tree models.
[16]	Refined weighted Random Forest (assigning different weights to different decision trees).	Better prediction power in comparison to standard random forests due to the following: (1) all training data including in-bag data and Out-of-Bag data is used and (2) the margin between probability of predicting true class and false class label applied.
[20]	Optimality conditions for four combination methods: majority vote (MV), weighted majority vote (WMV), the recall combiner (REC) and Naive Bayes (NB).	Experiments revealed that there is no dominant combiner. NB was the most successful but the differences with MV and WMV were not found to be statistically significant.
[22]	Weighting each tree by replacing the regular average with a Cesaro average (CRF—Cesaro Random Forest).	Although the Cesaro random forest appears to be competitive to the classical RF, it has limitations i.e., the way to determine the sequencing of trees (what impacts the results) and the probability estimates of class membership are not available.
[23]	Variable performance-weighted and Recency-weighted random forests.	The results show that recency-weighted ensembles of random forests produce superior results in terms of both profitability and prediction accuracy compared with other ensemble techniques.
[24]	Weighted random survival forest by assigning weights to survival decision trees or to their subsets.	Numerical examples with real data illustrate the outperformance of the proposed model in comparison with the original random survival forest.

**Table 2.** Methods of detecting arrhythmias.

Arrhythmia/Complex	Method	Work
QRS Detection	Pan-Tompkins (filtering techniques); Threshold-based detection; Multimodal data methods; Gradient calculations; Based on Peak energy; Markov-model; RS Slope detection; Low-complexity R-peak detector.	[26–35]
Asystole	Short term autocorrelation analysis; Flat line artefacts definition; Frequency domain analysis; Signal quality based rules.	[35–37]
Bradycardia and Tachycardia	Threshold +Support vector machine; Beat-to-beat Correlogram 2D.	[35,36]
Ventricular Tachycardia	Time-frequency representation images; Spectral characteristics of ECG; Spectra purity index; Autocorrelation function.	[31,36,38–44]
Ventricular Flutter or Fibrillation	Autocorrelation analysis; Wavelet transformations; Sample entropy; Machine learning methods with features derived from signal morphology and analysis of power spectrum; Time-frequency representation images; Empirical mode decomposition; The zero crossing rate combined with base noise suppression with discrete cosine transform and beat-to-beat intervals.	[39,42,43,45–49]
All types	Rule based methods; Regular-activity test; Single- and multichannel fusion rules; Machine learning algorithms; SVM—Support Vector Machines; LDA—Linear discriminant analysis; Random Forest classifiers.	[27,35,38,50,51]

**Table 3.** An example of weights deriving.

Tree No.	AUC_INB_	AUC_OOB_	Equation (4) (*α*=0.5)	Ranking	Nominator (*p* = 2)	Final We ights
1	0.70	0.70	0.350	3	4	0.133
2	0.65	0.55	0.325	4	1	0.034
3	0.90	0.80	0.450	1	16	0.533
4	0.85	0.80	0.425	2	9	0.300

**Table 4.** Improvement of Area Under the Curve (AUC) and Score for Asystole on validation sample in terms of the *α*-parameter and *p*-parameter.

Base		0.0	0.5	1.0	1.5	2.0	2.5	3.0	3.5	4.0	4.5	5.0
AUC = 0.93	**0**	0.000	0.055	0.055	0.055	0.055	0.055	0.055	0.055	0.055	0.050	0.050
	**0.1**	0.000	0.055	0.055	0.055	0.055	0.055	0.055	0.055	0.055	0.055	0.050
	**0.2**	0.000	0.055	0.055	0.055	0.055	0.055	0.055	0.055	0.055	0.055	0.050
	**0.3**	0.000	0.055	0.055	0.055	0.055	0.055	0.055	0.055	0.055	0.055	0.050
	**0.4**	0.000	0.055	0.055	0.055	0.055	0.055	0.055	0.055	0.055	0.055	0.050
	**0.5**	0.000	0.055	0.055	0.055	0.055	0.055	0.055	0.055	0.055	0.055	0.050
	**0.6**	0.000	0.055	0.055	0.055	0.055	0.055	0.055	0.055	0.055	0.055	0.050
	**0.7**	0.000	0.055	0.055	0.055	0.055	0.055	0.055	0.055	0.055	0.055	0.055
	**0.8**	0.000	0.055	0.055	0.055	0.055	0.055	0.055	0.055	0.055	0.055	0.055
	**0.9**	0.000	0.055	0.055	0.055	0.055	0.055	0.055	0.055	0.055	0.060	0.060
	**1**	0.000	0.055	0.055	0.055	0.055	0.055	0.055	0.060	0.060	0.060	0.060

SCORE = 61.75	**0**	0.00	19.00	19.00	19.00	19.00	19.00	19.00	19.00	19.00	19.00	19.00
	**0.1**	0.00	19.00	19.00	19.00	19.00	19.00	19.00	19.00	19.00	19.00	19.00
	**0.2**	0.00	19.00	19.00	19.00	19.00	19.00	19.00	19.00	19.00	19.00	19.00
	**0.3**	0.00	19.00	19.00	19.00	19.00	19.00	19.00	19.00	19.00	19.00	19.00
	**0.4**	0.00	19.00	19.00	19.00	19.00	19.00	19.00	19.00	19.00	19.00	19.00
	**0.5**	0.00	19.00	19.00	19.00	19.00	19.00	19.00	19.00	19.00	19.00	19.00
	**0.6**	0.00	19.00	19.00	19.00	19.00	19.00	19.00	19.00	19.00	19.00	19.00
	**0.7**	0.00	19.00	19.00	19.00	19.00	19.00	19.00	19.00	19.00	19.00	19.00
	**0.8**	0.00	19.00	19.00	19.00	19.00	19.00	19.00	19.00	19.00	19.00	19.00
	**0.9**	0.00	19.00	19.00	19.00	19.00	19.00	19.00	19.00	19.00	19.00	19.00
	**1**	0.00	19.00	19.00	19.00	19.00	19.00	19.00	19.00	19.00	19.00	19.00

**Table 5.** Improvement of AUC and Score for Bradycardia on validation sample in terms of the *α*-parameter and *p*-parameter.

Base		0.0	0.5	1.0	1.5	2.0	2.5	3.0	3.5	4.0	4.5	5.0
AUC = 0.95	**0**	0.000	0.000	−0.005	−0.011	−0.011	−0.011	−0.011	−0.011	−0.011	−0.011	−0.011
	**0.1**	0.000	0.000	−0.005	−0.011	−0.011	−0.011	−0.011	−0.011	−0.011	−0.011	−0.011
	**0.2**	0.000	0.000	−0.005	−0.005	−0.011	−0.011	−0.011	−0.011	−0.011	−0.011	−0.011
	**0.3**	0.000	0.001	−0.005	−0.005	−0.011	−0.011	−0.011	−0.011	−0.011	−0.011	−0.011
	**0.4**	0.000	0.001	−0.005	−0.005	−0.005	−0.011	−0.011	−0.011	−0.005	−0.005	−0.005
	**0.5**	0.000	0.001	−0.005	−0.005	−0.005	−0.005	−0.005	−0.005	−0.005	−0.005	−0.005
	**0.6**	0.000	0.001	−0.005	−0.005	−0.005	−0.005	−0.005	−0.005	−0.005	−0.005	−0.005
	**0.7**	0.000	0.001	−0.005	−0.005	−0.005	−0.005	−0.005	−0.005	−0.005	−0.005	−0.005
	**0.8**	0.000	0.001	0.001	−0.005	−0.005	−0.005	−0.005	−0.005	−0.005	−0.005	−0.005
	0.9	0.000	0.001	0.001	0.001	−0.005	−0.005	−0.005	−0.005	−0.005	−0.005	−0.005
	**1**	0.000	0.001	0.001	0.001	0.001	−0.005	−0.005	−0.005	−0.005	−0.005	−0.005

SCORE = 77.73	**0**	0.00	−0.02	−0.02	−0.02	−0.02	−1.27	−1.27	−1.27	−1.27	−1.27	−1.27
	**0.1**	0.00	−0.02	−0.02	−0.02	−0.02	−0.02	−1.27	−1.27	−1.27	−1.27	−1.27
	**0.2**	0.00	−0.02	−0.02	−0.02	−0.02	−0.02	−1.27	−1.27	−1.27	−1.27	−1.27
	**0.3**	0.00	−0.02	−0.02	−0.02	−0.02	−0.02	−0.02	−1.27	−1.27	−1.27	−1.27
	**0.4**	0.00	−0.02	−0.02	−0.02	−0.02	−0.02	−0.02	−0.02	−1.27	−1.27	−1.27
	**0.5**	0.00	−0.02	−0.02	−0.02	−0.02	−0.02	−0.02	−0.02	−0.02	−0.02	−1.27
	**0.6**	0.00	−0.02	−0.02	−0.02	−0.02	−0.02	−0.02	−0.02	−0.02	−0.02	−0.02
	**0.7**	0.00	−0.02	−0.02	−0.02	−0.02	−0.02	−0.02	−0.02	−0.02	−0.02	−0.02
	**0.8**	0.00	−0.02	−0.02	−0.02	−0.02	−0.02	−0.02	−0.02	−0.02	−0.02	−0.02
	**0.9**	0.00	−0.02	−0.02	−0.02	−0.02	−0.02	−0.02	−0.02	−0.02	−0.02	−0.02
	**1**	0.00	−0.02	−0.02	−0.02	−0.02	−0.02	−0.02	−0.02	−0.02	−0.02	−0.02

**Table 6.** Improvement of AUC and Score for Tachycardia on validation sample in terms of the *α*-parameter and *p*-parameter.

Base		0.0	0.5	1.0	1.5	2.0	2.5	3.0	3.5	4.0	4.5	5.0
AUC = 0.99	**0**	0.000	0.000	0.000	0.000	0.000	0.000	0.000	0.000	0.000	0.000	0.000
	**0.1**	0.000	0.000	0.000	0.000	0.000	−0.008	−0.008	−0.008	−0.008	−0.008	−0.008
	**0.2**	0.000	0.000	0.000	0.000	0.000	−0.008	−0.008	−0.008	−0.008	−0.008	−0.008
	**0.3**	0.000	0.000	0.000	0.000	0.000	0.000	−0.008	−0.008	−0.008	−0.008	−0.008
	**0.4**	0.000	0.000	0.000	0.000	0.000	0.000	−0.008	−0.008	−0.008	−0.008	−0.008
	**0.5**	0.000	0.000	0.000	0.000	0.000	0.000	−0.008	−0.008	−0.008	−0.001	−0.001
	**0.6**	0.000	0.000	0.000	0.000	0.000	0.000	−0.008	−0.008	−0.008	−0.001	−0.001
	**0.7**	0.000	0.000	0.000	0.000	0.000	0.000	0.000	−0.008	−0.008	−0.008	−0.001
	**0.8**	0.000	0.000	0.000	0.000	0.000	0.000	0.000	−0.008	−0.008	−0.008	−0.008
	**0.9**	0.000	0.000	0.000	0.000	0.000	0.000	0.000	−0.008	−0.008	−0.008	−0.008
	**1**	0.000	0.000	0.000	0.000	0.000	0.000	0.000	0.000	−0.008	−0.008	−0.008

SCORE = 81.08	**0**	0.00	0.00	0.00	0.00	0.00	0.00	0.00	0.00	0.00	0.00	0.00
	**0.1**	0.00	0.00	0.00	0.00	0.00	0.00	0.00	0.00	0.00	0.00	0.00
	**0.2**	0.00	0.00	0.00	0.00	0.00	0.00	0.00	0.00	0.00	0.00	0.00
	**0.3**	0.00	0.00	0.00	0.00	0.00	0.00	0.00	0.00	0.00	0.00	0.00
	**0.4**	0.00	0.00	0.00	0.00	0.00	0.00	0.00	0.00	0.00	0.00	0.00
	**0.5**	0.00	0.00	0.00	0.00	0.00	0.00	0.00	0.00	0.00	0.00	0.00
	**0.6**	0.00	0.00	0.00	0.00	0.00	0.00	0.00	0.00	0.00	0.00	−9.25
	**0.7**	0.00	0.00	0.00	0.00	0.00	0.00	0.00	0.00	0.00	0.00	0.00
	**0.8**	0.00	0.00	0.00	0.00	0.00	0.00	0.00	0.00	0.00	0.00	0.00
	**0.9**	0.00	0.00	0.00	0.00	0.00	0.00	0.00	0.00	0.00	0.00	0.00
	**1**	0.00	0.00	0.00	0.00	0.00	0.00	0.00	0.00	0.00	0.00	0.00

**Table 7.** Improvement of AUC and Score for Ventricular Fibrillation or Flutter on validation sample in terms of the *α*-parameter and *p*-parameter.

Base		0.0	0.5	1.0	1.5	2.0	2.5	3.0	3.5	4.0	4.5	5.0
AUC = 0.97	**0**	0.000	0.030	0.030	0.030	0.030	0.030	0.030	0.030	0.030	0.030	0.030
	**0.1**	0.000	0.030	0.030	0.030	0.030	0.030	0.030	0.030	0.030	0.030	0.030
	**0.2**	0.000	0.030	0.030	0.030	0.030	0.030	0.030	0.030	0.030	0.030	0.030
	**0.3**	0.000	0.030	0.030	0.030	0.030	0.030	0.030	0.030	0.030	0.030	0.030
	**0.4**	0.000	0.030	0.030	0.030	0.030	0.030	0.030	0.030	0.030	0.030	0.030
	**0.5**	0.000	0.030	0.030	0.030	0.030	0.030	0.030	0.030	0.030	0.030	0.030
	**0.6**	0.000	0.030	0.030	0.030	0.030	0.030	0.030	0.030	0.030	0.030	0.030
	**0.7**	0.000	0.030	0.030	0.030	0.030	0.030	0.030	0.030	0.030	0.030	0.030
	**0.8**	0.000	0.030	0.030	0.030	0.030	0.030	0.030	0.030	0.030	0.030	0.030
	**0.9**	0.000	0.030	0.030	0.030	0.030	0.030	0.030	0.030	0.030	0.030	0.030
	**1**	0.000	0.030	0.030	0.030	0.030	0.030	0.030	0.030	0.030	0.030	0.009

SCORE = 30.56	**0**	0.00	55.55	55.55	55.55	55.55	55.55	55.55	55.55	55.55	55.55	55.55
	**0.1**	0.00	55.55	55.55	55.55	55.55	55.55	55.55	55.55	54.51	54.51	54.51
	**0.2**	0.00	55.55	55.55	55.55	55.55	55.55	55.55	55.55	54.51	54.51	54.51
	**0.3**	0.00	55.55	55.55	55.55	55.55	55.55	55.55	55.55	54.51	54.51	54.51
	**0.4**	0.00	55.55	55.55	55.55	55.55	55.55	55.55	55.55	54.51	54.51	54.51
	**0.5**	0.00	55.55	55.55	55.55	55.55	55.55	55.55	55.55	54.51	54.51	54.51
	**0.6**	0.00	55.55	55.55	55.55	55.55	55.55	55.55	55.55	54.51	54.51	54.51
	**0.7**	0.00	55.55	55.55	55.55	55.55	55.55	55.55	55.55	54.51	54.51	54.51
	**0.8**	0.00	55.55	55.55	55.55	55.55	55.55	55.55	55.55	54.51	54.51	54.51
	**0.9**	0.00	55.55	55.55	55.55	55.55	55.55	55.55	55.55	54.51	54.51	54.51
	**1**	0.00	55.55	55.55	55.55	55.55	55.55	55.55	55.55	54.51	54.51	54.51

**Table 8.** Improvement of AUC and Score for Ventricular Tachycardia on validation sample in terms of the *α*-parameter and *p*-parameter.

Base		0.0	0.5	1.0	1.5	2.0	2.5	3.0	3.5	4.0	4.5	5.0
AUC = 0.87	**0**	0.000	−0.001	0.000	0.003	0.001	0.002	0.001	0.001	0.001	0.001	0.003
	**0.1**	0.000	−0.001	0.000	0.002	0.002	0.002	0.002	0.002	0.001	0.000	0.002
	**0.2**	0.000	−0.002	0.000	0.001	0.002	0.001	0.001	0.001	0.001	0.001	0.002
	**0.3**	0.000	−0.002	0.001	0.001	0.002	0.002	0.001	0.001	0.001	0.001	0.003
	**0.4**	0.000	−0.002	0.001	0.000	0.002	0.002	0.001	0.001	0.000	0.001	0.003
	**0.5**	0.000	−0.002	0.001	0.000	0.001	0.002	0.002	0.002	0.001	0.001	0.002
	**0.6**	0.000	−0.002	0.001	0.000	0.001	0.002	0.002	0.001	0.002	0.002	0.003
	**0.7**	0.000	−0.002	0.000	0.000	0.001	0.002	0.002	0.002	0.001	0.001	0.003
	**0.8**	0.000	−0.002	0.001	0.000	0.001	0.001	0.002	0.002	0.001	0.003	0.002
	**0.9**	0.000	−0.002	0.000	0.001	0.001	0.001	0.002	0.001	0.000	0.002	0.002
	**1**	0.000	−0.002	−0.001	0.000	0.001	0.001	0.002	0.001	0.000	0.001	0.001

SCORE = 31.54	**0**	0.00	−0.26	1.11	0.42	0.42	0.42	0.31	0.31	0.21	0.21	0.21
	**0.1**	0.00	−0.26	1.11	0.42	0.42	0.42	0.31	0.21	0.21	0.21	0.21
	**0.2**	0.00	−0.26	1.11	0.42	0.42	0.42	0.42	0.42	0.31	0.21	0.21
	**0.3**	0.00	−0.26	1.11	0.42	0.42	0.42	0.42	0.42	0.31	0.31	0.31
	**0.4**	0.00	−0.26	1.11	0.42	0.42	0.42	0.42	0.31	0.31	0.31	0.31
	**0.5**	0.00	−0.26	1.11	0.42	0.42	0.42	0.42	0.42	0.31	0.31	0.31
	**0.6**	0.00	−0.26	1.11	0.42	0.42	0.42	0.42	0.31	0.31	0.31	0.31
	**0.7**	0.00	−0.26	1.11	0.42	0.42	0.42	0.42	0.31	1.01	1.01	0.33
	**0.8**	0.00	−0.26	1.11	0.42	0.42	0.42	0.42	1.01	1.01	0.33	0.33
	**0.9**	0.00	−0.26	0.44	0.42	0.42	0.42	1.11	1.01	0.33	0.33	0.33
	**1**	0.00	−0.26	0.44	0.42	0.42	1.11	1.11	0.33	0.33	0.23	0.23

**Table 9.** Detailed classification results for benchmarking methods.

Arrhythmia Type	Method	AUC	Score
Asystole	Weighted RF (*α* = 0.9, *p* = 4.5) Standard RF CART (*cp* = 0.065) C-SVM (polynomial = 2, *ε* = 0.3, *C* = 0.4) AdaBoost	(98.5 ± 3.1) (92.5 ± 3.5) (86.0 ± 4.2) (92.0 ± 3.7) (91.9 ± 3.9)	(80.7 ± 8.7) (61.7 ± 9.2) (52.1 ± 10.9) (61.7 ± 9.2) (61.7 ± 9.2)
Extreme Bradycardia	Weighted RF (*α* = 0.5, *p* = 0.5) Standard RF CART (*cp* = 0.083) C-SVM (polynomial = 2, *ε* = 0.3, *C* = 0.4) AdaBoost	(95.6 ± 4.4) (95.0 ± 4.5) (87.5 ± 4.4) (95.2 ± 4.5) (95.1 ± 4.6)	(77.7 ± 9.7) (77.7 ± 9.7) (63.1 ± 10.5) (77.7 ± 9.7) (77.7 ± 9.7)
Ventricular Tachycardia	Weighted RF (*α* = 0.7, *p* = 5.0) Standard RF CART (*cp* = 0.011) C-SVM (radial = 0.1, *ε* = 0.1, *C* = 0.8) AdaBoost	(87.5 ± 3.5) (87.3 ± 3.5) (72.6 ± 4.2) (86.1 ± 3.7) (83.1 ± 3.9)	(31.9 ± 2.7) (31.5 ± 2.7) (27.7 ± 4.2) (30.5 ± 3.7) (29.9 ± 3.7)
Ventricular Fibrillation or Flutter	Weighted RF (*α* = 0.5, *p* = 1.0) Standard RF CART (*cp* = 0.017) C-SVM (radial = 0.5, *ε* = 0.1, *C* = 0.8) AdaBoost	(99.9 ± 0.1) (97.0 ± 2.1) (89.9 ± 8.7) (97.5 ± 5.3) (97.5 ± 5.3)	(86.1 ± 7.7) (30.6 ± 13.9) (29.4 ± 16.6) (50.1 ± 12.8) (50.1 ± 12.8)
Extreme Tachycardia	Weighted RF (*α* = 0.1, *p* = 1.0) Standard RF CART (*cp* = 0.090) C-SVM (polynomial = 3, *ε* = 0.5, *C* = 0.6) AdaBoost	(99.2 ± 0.1) (99.2 ± 0.1) (64.2 ± 8.6) (99.2 ± 0.1) (99.2 ± 0.1)	(81.1 ± 7.7) (81.1 ± 7.7) (53.6 ± 9.9) (81.1 ± 7.7) (81.1 ± 7.7)
